# Vaping-Associated Lung Injury: A Review

**DOI:** 10.3390/medicina58030412

**Published:** 2022-03-10

**Authors:** Marissa O’Callaghan, Niamh Boyle, Aurelie Fabre, Michael P. Keane, Cormac McCarthy

**Affiliations:** 1 Department of Respiratory Medicine, St. Vincent’s University Hospital, D04 T6F Dublin, Ireland; marissa.ocallaghan1@ucd.ie (M.O.); niamhpboyle@svhg.ie (N.B.); michael.p.keane@ucd.ie (M.P.K.); 2 School of Medicine, University College Dublin, D14 E099 Dublin, Ireland; afabre@svhg.ie; 3 Department of Histopathology, St. Vincent’s University Hospital, D04 T6F Dublin, Ireland

**Keywords:** vaping, EVALI, e-cigarette, foamy macrophages, Oil Red O stain

## Abstract

Since commercial development in 2003, the usage of modern electronic cigarette (e-cigarette) continues to increase amongst people who have never smoked, ex-smokers who have switched to e-cigarettes, and dual-users of both conventional cigarettes and e-cigarettes. With such an increase in use, knowledge of the irritative, toxic and potential carcinogenic effects on the lungs is increasing. This review article will discuss the background of e-cigarettes, vaping devices and explore their popularity. We will further summarise the available literature describing the mechanism of lung injury caused by e-cigarette or vaping use.

## 1. Introduction

The lungs are exposed to a multitude of environmental agents with each inspiration. Some of these agents are toxic or cause damage to the lungs. Vaping or electronic cigarette (e-cigarette) use is no exception. These devices aerosolise a liquid vapour, which is then inhaled. This vapour contains chemical compounds such as nicotine, flavourings and tetrahydrocannabinol (THC). Some of these chemicals have irritative, toxic and carcinogenic properties [1,2,3,4]. When inhaled these can alter the immune responses critical for normal lung function and cause lung injury. The pathological manifestation of this is diverse varying from organising pneumonia or diffuse alveolar damage to established interstitial lung disease (ILD) [5,6,7,8,9]. Originally marketed as a harm reduction tool for cigarette smokers [10], the number of people vaping continues to rise despite vaping related lung injury being increasingly recognised [11]. Here, we review the origins of e-cigarette use, the factors contributing to increased use and the immune changes that occur within the lungs following exposure and elucidate the proposed mechanisms implicated in vaping related lung injury.

## 2. Materials and Methods

For this review, we took a systematic approach to identify all of the peer-reviewed studies concerning the impact of e-cigarette and vaping use on the lungs. A bibliographic search was performed on MEDLINE on 16 October 2021, using the following keywords: (EVALI OR vaping OR e-cigarette OR VALI OR vaping associated lung injury *). We then searched for additional relevant papers after reviewing the works cited in the papers identified and through the “relevant articles” section on MEDLINE. We included papers that included case series and a selection of case reports in Table 1.

## 3. Background

The damaging health consequences of cigarette smoking have been widely acknowledged for years [33]. While the ability to diagnose and the management of smoking-related diseases has improved considerably, cigarette smoking, unfortunately, remains prevalent, so health services remain inundated with smoking-related illnesses [34,35]. These include, but are not limited to, coronary artery disease, cerebrovascular disease, chronic obstructive pulmonary disease, lung cancer and respiratory tract infections [36,37]. There remains some hope for the future, however, as smoking rates in most Organisation for Economic Co-operation and Development (OECD) countries have decreased over the last decade, from an average of 23% in 2007 to 18% in 2017 [38]. There is a wealth of evidence that successful smoking cessation improves mortality, regardless of age at cessation [35,39]. Programmes to prevent and treat tobacco dependence are abundant and have demonstrated considerable, albeit insufficient, success. There is good evidence to support the role of several nicotine replacement therapies, delivered in various forms such as transdermal patches, gum and lozenges, in smoking reduction and cessation [35,40,41,42,43]. There are also non-nicotine pharmacotherapies and psychosocial interventions that are widely available and help with smoking cessation [35,44].

**Figure 1:**
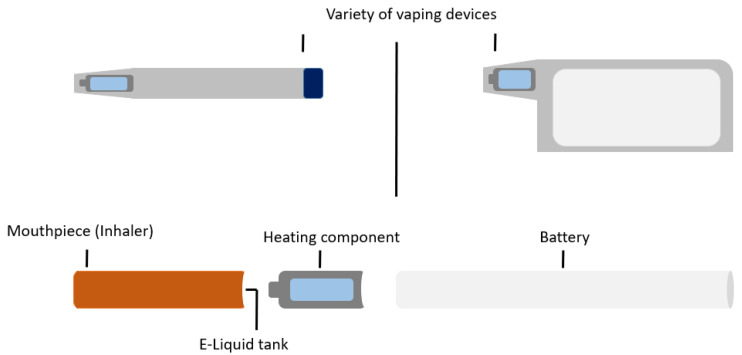
E-cigarette or vaping device.

Modern e-cigarettes, commercially developed in 2003, were advertised as a novel therapy for smoking cessation [11]. These electronic devices are designed to vaporise chemical compounds [45], the term ‘vaping’ referring to the perception that the exhaled smoke is water vapour. It actually consists of fine particles of chemicals mixed in vegetable glycerin (VG) and/or propylene glycol (PG) [46]. The vaping device consists of a mouthpiece, a battery, a tank which contains the “e-liquid” or “e-juice” and a heating component for the device (Figure 1) [45,47].

E-cigarettes have since been developed in various shapes, sizes and device types. Different terminologies used to describe these devices include e-cigs, vapes, e-hookahs, vape pens, mods, tanks or electronic nicotine delivery systems (ENDS) [45]. All delivery devices work on a similar principle. Electricity, activated manually or automatically by a battery, is delivered to the device’s heating component. This, in turn, causes the e-liquid contained in the tank to evaporate and condense into a fine mist of liquid droplets (aerosols) [48]. The e-liquid or substance placed in the device or tank is user-dependant. Commonly used substances include nicotine, fruity and menthol flavouring. A minority of users use e-liquids from unauthorised sources or modify the e-liquid contents. This risks exposure to potentially harmful compounds such as heavy metals or carcinogenic chemicals [45,49].

## 4. Epidemiology of E-Cigarette Use

E-Cigarettes are marketed as a harm reduction tool for tobacco smokers wishing to quit [11]. They are advertised as a safe and viable alternative to cigarette smoking; however, there is a lack of evidence to prove superiority to conventional smoking cessation strategies [45,50]. E-cigarette use worldwide has grown dramatically, with a prevalence of 5.5% amongst adults in both North America and England [11,51,52,53]. The response from tobacco regulatory officials has been mixed. Some are promoting the use of e-cigarettes for harm minimisation [10], while others have requested regulation of the nicotine market, favouring proven smoking cessation techniques. Data to support any of these marketing strategies remain limited. E-Cigarette users are varied and include people who have never smoked, ex-smokers who have switched to e-cigarettes and dual-users of both conventional cigarettes and e-cigarettes [11]. The role of e-cigarettes as a smoking cessation tool is hotly contested. Trial results have been mixed and ultimately inconclusive. E-cigarettes with nicotine likely increase smoking cessation rates compared to e-cigarettes without nicotine. In addition, there is no clear evidence of harm from nicotine e-cigarettes; however, the patient numbers in the studies to date have been low, and the longest follow-up period was two years [54,55]. E-cigarettes with nicotine demonstrated improved smoking cessation rates over conventional nicotine replacement therapy in a recently published randomised control trial. The caveat in this study was that participants in both groups had regular face-to-face meetings with clinicians, a form of support that is rarely provided to those seeking to quit in the real world [56]. Furthermore, only 18% of participants in the e-cigarette group stopped smoking entirely, suggesting that e-cigarettes are a far cry from a “cure” for tobacco smoking. Some studies even suggest that there are increased smoking relapse rates when e-cigarettes are used as a cessation tool [57,58].

A most worrying trend has been noticed amongst adolescents with e-cigarette use, especially flavoured e-cigarettes, increasing rapidly over time [35,59]. We know that e-cigarettes may encourage initiation of conventional cigarettes among non-smokers, and with e-cigarette use rising from 4.7 to 10.0% in one study of high school students, there is a good reason for concern [35,60]. Reports show that appealing flavours are one of the principal reasons for e-cigarette use amongst adolescents and young adults [59,61,62]. Flavourings may make the use of e-cigarettes more desirable and enjoyable [63], especially if sweet-flavoured. Users are more likely to use again and place perceived monetary value on sweet-flavoured versus non-sweet or unflavoured e-cigarettes [59,64].

Major tobacco companies entered the e-cigarette industry in 2012 and have since progressively dominated the market, buying out smaller retailers [65,66]. These companies are more likely to sell ‘cigalikes’, a form of e-cigarette with a slim cylindrical closed-system design that uses prefilled cartridges, to maximise the ease of use. These devices mimic the experience of smoking conventional cigarettes and studies suggest that smokers of cigalikes are more likely to remain dual users [66,67,68]. While other factors may also be contributory, international surveys show that one out of eight smokers have tried e-cigarettes, with the highest prevalence being amongst younger, female, higher-income smokers [69,70]. E-cigarette users view e-cigarettes as safer, healthier and less likely to cause dependency than conventional cigarettes [35,69,70,71,72,73,74]. However, without clear evidence of a role in the reduction of tobacco dependence, e-cigarettes risk renormalising and re-glamorising smoking. This is of paramount concern, potentially undoing years of effort by the public health and medical communities [35,75].

## 5. Mechanism of Injury with Vaping

While analysis of e-cigarette efficacy in aiding smoking cessation is ongoing, data on the overall impact of e-cigarettes on population health is limited [35]. Over 7000 compounds and at least 70 carcinogens have been identified in conventional tobacco smoke [33,35,76,77,78]. Studies comparing the toxic exposures between e-cigarettes and conventional cigarettes reported that levels of two nitrosamines and carbon monoxide were lower in e-cigarette users than in smokers but present nonetheless [1,2,3,4]. Unsurprisingly, toxic exposures were greatest in dual users [78]. Both smokers and e-cigarette users also had increased toxic metals in urine and blood, but there was some variability in the metals detected in each group [1,4,11,78].

Studies such as these prompted the Food and Drug Administration (FDA) to release a warning regarding the potential health risks of e-cigarettes in 2009 [35,79]. While there are greater chemical emissions from combustible tobacco cigarette smoke than from most e-cigarette products, the chemicals in e-liquids and the additional chemicals generated during the aerosolisation of e-liquids also have potential toxic properties [80,81,82]. It has been proposed that oxidative stress is the primary driver of e-cigarette-induced toxicity at a cellular level [11,80,83,84,85,86,87,88,89,90]. While this theory is a plausible explanation for the tissue injuries reported in the literature, the impact is less than that caused by combustible tobacco smoking [91]. Another group demonstrated acute endothelial cell dysfunction following e-cigarette aerosol exposure but highlights the uncertainty surrounding the long-term consequences and outcomes with long term exposure [80,92]. 

Scott et al. sought to replicate the potential effects of exposure of the e-cigarette user in an acute in vitro system using a vaping-condensate technique. They showed that exposure of macrophages to e-cigarette vapour-condensate induced many of the same cellular and functional changes in alveolar macrophages seen in cigarette smokers and patients with COPD [83]. Adolescents who use e-cigarettes commonly report an increased cough and wheeze, and studies have shown an association with e-cigarette use and asthma exacerbations [34,93,94]. However, it is not yet clear if chronic e-cigarette use by itself can cause COPD in a clinical setting or if the substitution of e-cigarettes for combustible tobacco products can prevent or slow the development of COPD [80,95,96]. There is also data to support a correlation between e-cigarette use and impaired host defence [97,98]. It appears viral responses are compromised [86], and bacterial clearance by macrophages [83,99] and neutrophils appears to be reduced [100,101]. This allows increased adhesion and colonisation of bacteria [100,102] and possibly an impaired infection-fighting ability [35]. 

Many groups have also studied the impact of e-cigarette use on lung function [103,104,105]. One study looked at the impact of theatrical smokes and fogs, which have similar additives to the liquid nicotine cartridge (glycol derivatives), on lung function [106,107]. A total of 27 healthy people without asthma were exposed to propylene glycol for 1 min. This resulted in a 2% reduction in FEV1/FVC (*p* = 0.049), a 40 mL increase in FVC (*p* = 0.23) and a 30 mL fall in FEV1 (*p* = 0.29) [107]. In a separate study, the lung function of 101 staff members working at sites using theatrical fog (usually working < 10 feet from fog-generating machines) was measured. A 5% reduction in FEV1 and FVC was noted in comparison to staff working further away [106]. While this data cannot be generalised to e-cigarette users, it highlights the acute effects of vapours similar to those from e-cigarettes [35].

## 6. Electronic Cigarette and Vaping-Associated Lung Injury: EVALI

Since their introduction, there have been a number of case reports published describing a link between e-cigarette use and respiratory disease [5,6,7,8,9,28]. In the United States in 2019, there was a large number of lung injuries reported associated with e-cigarette use. This disease entity was subsequently labelled as EVALI (e-cigarette and vaping-associated lung injury). The initial reports were of a small number of patients in two states with lung injuries associated with the use of e-cigarettes in the days and weeks before symptom onset. Subsequently, an unprecedented epidemic of lung injury was reported across all states in the US, with more than 2500 cases reported nationwide within a 5 month period [9,108].

**Figure 2:**
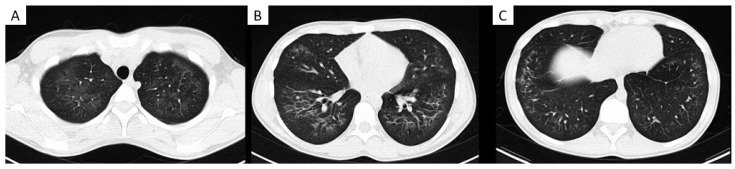
Images show electronic cigarette or vaping product use–associated lung injury in an 18-year-old male who attended our institution. Axial CT chest imaging (**A**–**C**) demonstrates extensive bilateral centrilobular and peri-bronchial ground glass opacification with subpleural sparing, slightly more confluent in the lower zones.

A wide range of clinical presentations have been reported in the literature [7,8,9,13,15,21,22,23,28]. Respiratory symptoms are the most prevalent at hospital presentation, specifically dyspnoea, cough and chest pain. Many patients report associated gastrointestinal symptoms and constitutional symptoms, most commonly subjective fever [9,13] (Table 1). Laboratory testing frequently reveals a peripheral blood eosinophilia, elevated erythrocyte sedimentation rate (ESR) and the presence of lipid-laden macrophages on bronchoalveolar lavage (BAL) assessment [21,23,109]. Most patients will have abnormal chest imaging. Typical computed tomography (CT) thorax findings are bilateral lung opacities with ground-glass changes, sometimes with subpleural sparing (Figure 2). Other reported findings include pneumomediastinum, pleural effusion and pneumothorax [9].

Although the exact mechanism of lung injury remains unclear and under investigation, exposure to products containing tetrahydrocannabinol (THC) was reported in over 80% of cases [110], and in many cases, unregulated or illicit street sources of THC were reported [111,112]. THC is the main psychoactive component in cannabis and despite THC-based oils and waxes being illegal in most American States, they remain easily accessible. THC-containing products that were seized by United States law enforcement at the peak of the EVALI outbreak, contained higher levels of vitamin E acetate than would be expected [9,113]. Furthermore, BAL samples from patients with EVALI have shown high rates of vitamin E [114] with a notable absence of vitamin E acetate in samples obtained from a healthy comparison group [115]. Vitamin E is a naturally occurring compound in surfactant, however, in contrast, vitamin E acetate is the synthetic ester of tocopherol and acetate. It is commonly added to e-liquids as a thickening agent [115].

**Figure 3:**
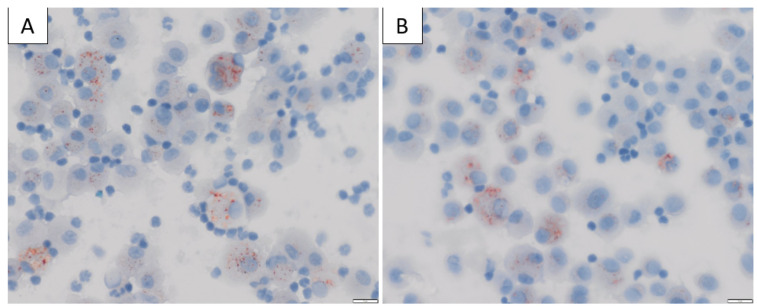
(**A**,**B**) Bronchoalveolar lavage (BAL) cytology from a patient diagnosed with EVALI in our institution, stained with Oil-Red-O × 400 magnification showing positive red intracytoplasmic droplets in the alveolar macrophages, consistent with excess neutral lipid.

**Figure 4:**
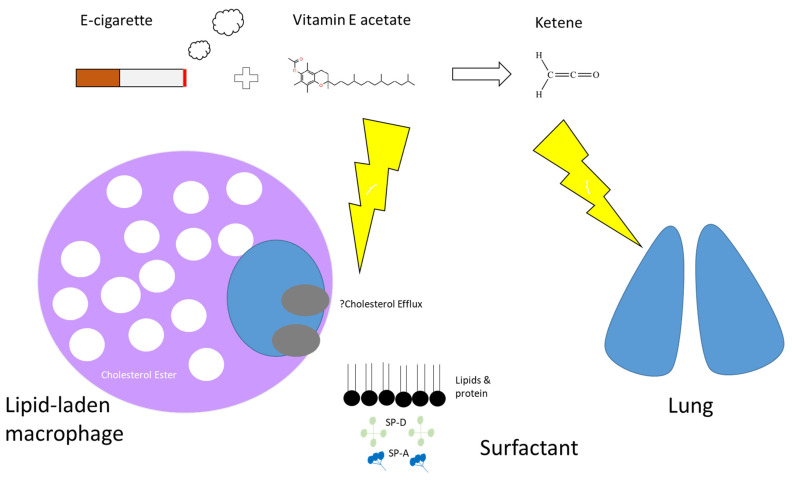
Proposed mechanism of action by which e-cigarettes cause lung injury. Many e-cigarettes that contain tetrahydrocannabinol (THC) have been shown to have higher levels of vitamin E acetate, commonly used as a thickening agent. It is possible that increased exposure of the lungs to Vitamin E (naturally occurring at low levels in surfactant) or Vitamin E acetate could affect the physical structure and phase behaviour of surfactant, impairing its ability to maintain surface tension leading to respiratory distress. Dysfunctional surfactant might lead to excess lipid accumulation within alveolar macrophages and that reverse cholesterol transport or cholesterol efflux might be implicated. Secondly, a known product of vaporised vitamin E acetate is ketene which is believed to be a lung irritant.

The mechanism by which vitamin E acetate causes lung injury is not fully understood. Mice exposed to aerosols generated from vitamin E acetate have demonstrated elevated levels of BAL lipid-laden macrophages on Oil-Red-O stain [116], which is in keeping with the BAL findings in patients with EVALI [21,109] (Figure 3). The presence of lipid-laden macrophages led to studies looking at macrophage lipid metabolism. It is possible that increasing concentrations of vitamin E or vitamin E acetate could affect the physical structure and phase behaviour of surfactants [115,117]. This may then impair the ability of the surfactants to maintain alveolar surface tension, leading to respiratory dysfunction [28,115,116]. Furthermore, vitamin E acetate forms a toxic compound, ketene, when heated. Ketene is a known lung irritant and thus may also contribute to the chemical pneumonitis seen in patients with EVALI [115,118,119] (Figure 4).

While most patients with EVALI went on to have a full recovery, over 2800 patients were hospitalised, and 68 deaths were reported throughout the outbreak [120]. Many case reports describe improvement with corticosteroid therapy [15]; however, the natural progression of this injury is not yet known, and it is possible that patients might recover without steroids or by avoiding use of e-cigarettes alone [15]. Nevertheless, because the diagnosis remains one of exclusion, empiric antimicrobial therapy might be warranted for patients with severe illness [9,121].

Since the outbreak in 2019, case numbers have declined. This led the American centre for disease control (CDC) to stop collecting data on EVALI in early 2020. The reasons behind the decline in case numbers is likely a combination of increased public awareness of the risk associated with THC-containing e-cigarettes [112], the removal of vitamin E acetate from some products and enhanced law enforcement actions related to illicit products all playing a role [120]. More recently, the FDA has extended its Premarket Tobacco Product Application (PMTA) to e-cigarette and vaping products. This process began initially in 2016 but was deferred to allow retailers sufficient time to make their PMTA application and finally took effect as of September 2020. This ruling will ensure the standardisation and regulation of the contents in e-cigarette and vaping devices [122].

In spite of these advances, the high prevalence of youth and young adult use of nicotine and cannabis e-cigarettes remains a source of concern [123,124]. Cannabis policies are rapidly changing worldwide, and with this, the frequency of cannabis vaping rising. One study quoted a doubling of cannabis vaping frequency amongst high school seniors from 2018 to 2019 [123]. Youth vaping habits suggest that e-cigarettes are as popular as ever, and the fear is that cannabis vaping may act as a gateway to experimentation with vaping of illicit and black-market products, especially amongst curious high-school students and young adults. These users are not only at risk of harm from other potential mechanisms of e-cigarette-induced injury but also the added uncertainty around the consequences of vaporising unknown substances or chemicals.

## 7. Conclusions

E-cigarette use has grown exponentially, particularly amongst adolescents [34]. While they were originally marketed as a smoking cessation tool, evidence for a role in smoking cessation has been inconclusive. There is some evidence that nicotine-containing products may have a role in risk reduction for active smokers but, conversely, may encourage the initiation of cigarette smoking in non-smokers [55,56,57]. While the overall health effects of e-cigarettes are limited, pulmonary toxicity is established. At the cellular level, studies have shown increased oxidative stress, endothelial cell dysfunction and impaired host defence with functional changes in macrophages and neutrophils [80,81,82,83,84,85,86,87,88,89,90,91,100,101,102]. Clinically, lung injuries in the setting of exposure to vaping products, in particular THC, have been reported widely [9,110,125,126]; however, the long-term effects remain unknown. Studies will be required to further elucidate the multifaceted mechanisms underpinning the lung injury and the complex interaction between the inhalation of noxious factors and the host immune response in vaping-related illness.

## Figures and Tables

**Table 1:** Variation in clinical and radiological presentation of patients with EVALI.

Study	#	Symptoms	Vital Signs	Radiology (CT Chest Findings)	Laboratory Findings	BAL Findings	Clinical Course and Outcomes
Layden et al., 2020; USA [9]	98	**Respiratory** SOB 83/98 (85%) Chest pain 51/98 (52%) Cough 83/98 (85%) Hemoptysis 8/98 (8%) **GI** Nausea 65/98 (66%) Vomiting 60/98 (61%) Diarrhoea 43/98 (44%) Abdominal pain 33/98 (34%) **Constitutional** Fever 82/98 (84%) Weight loss 25/98 (26%) Fatigue 46/98 (34%)	Pyrexia 33% Hypoxia (SpO_2_ < 95%) 58%	Bilateral infiltrates 100%	ESR > 30 mm/h in 90%	2–68% macrophages 56% of those reported presence of LLMs	Admitted 93/98 Intubation 25/98 Antibiotics 86/93 Steroids 78/93 Died 2/98
Blagev et al., 2019; USA [12]	60	**Respiratory** SOB 51/60 (85%) Chest pain 26/60 (43%) Cough 47/60 (78%) Hemoptysis 7/60 (12%) **GI** Nausea 45/60 (75%) Vomiting 43/60 (72%) Abdominal pain 28/60 (47%) **Constitutional** Fever 46/60 (78%) Weight loss 7/60 (12%) Fatigue 29/60 (48%)	Pyrexia 57% Hypoxia (SpO_2_ < 95%) 87%	Abnormal Chest CT 100%	Mean CRP 31 mg/L ESR > 3.0 mm/h in 92%	63% (12/19) neutrophil predominant BAL21% (4/19) macrophage predominant BAL89% (8/9) reported presence of LLMs	Admitted 54/60 Intubation 10/60 Antibiotics 54/60 Steroids 57/60 Died 2/60
Zou et al., 2020; USA [13]	36	-	Mean fever 38.1 Mean SpO_2_ 94%	Abnormal 97%	-	-	Admitted 36/36 Intubation 7/36 Antibiotics 28/36 Steroids 26/36
Sangani et al., 2020; USA [14]	17	**Respiratory** SOB 17/17 (100%) Chest pain 6/17 (35%) Cough 12/17 (71%) **GI** symptoms 9/17 (53%) **Fever** 12/60 (71%) **Constitutional** symptoms 12/17 (71%)	-	Bilateral GGO 82% Consolidation 41%	-	24% (4/15) Neutrophil predominant BAL 87% (13/15) had ORO staining on BAL sample	Admitted 17/17 Intubation 7/17
Kalininskiy et al., 2019; USA [15]	12	**Respiratory** SOB 10/11 (91%) Cough 9/11 (82%) Pleuritic pain 6/11 (55%) Sputum 4/11 (36%) Haemoptysis 1/11 (9%) **GI** Vomiting 10/11 (91%) Nausea 7/11 (64%) Abdominal pain 3/11 (27%) Diarrhoea 3/11 (27%) **Constitutional** Fever 10/12 (83%) Malaise 9/12 (75%) Sweats 5/12 (42%) Myalgia 2/12 (17%)	Pyrexia 75% Hypoxia (SpO_2_ < 95%) 75%	Bilateral GGO 100% Subpleural sparing 64% Fibrotic features 18%	Median Eos 0.03 × 10^9^ Median CRP 232 mg/L Median ESR 80.5 mm/h	-	Admitted 12/12 Intubation 1/12 Antibiotics 11/12 Steroids 8/12
Doukas et al., 2020; USA [16]	10	**Respiratory** symptoms 80% (8/10) **GI** symptoms 60% (6/10) **Constitutional** symptoms 60% (6/10)	Pyrexia (40%) Hypoxia (SpO_2_ < 95%) 60%	Bilateral GGO 100%	-	-	Admitted 9/10 Antibiotics 10/10 IV steroids 4/10
Kass et al., 2020; USA [17]	10	**Respiratory** SOB 5/10 (50%) Cough 6/10 (60%) Pleuritic pain 3/10 (30%) Haemoptysis 3/10 (30%) **GI** Vomiting 5/10 (50%) Nausea 4/10 (40%) Diarrhoea 3/10 (30%) Weight loss 2/10 (20%) **Constitutional** Fever 3/10 (30%) Fatigue 1/10 (10%) Sweats 2/10 (20%)	Hypoxia (SpO_2_ < 95%) 60%	Bilateral GGO 50% Consolidation 10% Cavitation 10% Bronchiectasis 30%	Mean CRP (9/10) 8.93 mg/dL Mean ESR (4/10) 11.25 mm/h	6/10 patients 66% macrophage predominant BAL All with LLMs 16% neutrophil and eosinophilic predominant BAL	Admitted 10/10 Intubated 1/10 Antibiotics 10/10 Steroids 6/10
Kaous et al., 2020; USA [18]	8	**Respiratory** SOB 8/8 (100%) Chest pain 3/8 (37.5%) **GI** Nausea 4/8 (50%) Vomiting 1/8 (12.5%) **Constitutional** Fever 6/8 (75%) Myalgia 3/8 (37.5%)	Hypoxia (not defined) 87.5%	Bilateral opacities 100%	-	50% (3/6) Macrophage predominance on BAL 100% (6/6) LLMs on BAL	Admitted 8/8 Steroids 6/8
Corcoran et al., 2020; USA [19]	7	**Respiratory** SOB 5/7 (71%) Cough 6/7 (85%) Chest pain 3/7 (42%) Haemoptysis 1/7 (14%) **GI** Vomiting 5/7 (71%) **Constitutional** Fevers 4/7 (57%)	Pyrexia 42% Hypoxia (SpO_2_ < 95%) 57%	Bilateral GGO 85% Consolidation 42% Nodules 14%	Median CRP 34.9 mg/dL	-	Admitted 7/7 Antibiotics 6/7 Steroids 3/7
Khan et al., 2021; USA [20]	7	**Respiratory** SOB 5/7 (71%) Cough 6/7 (85%) Chest pain 2/7 (28%) **GI** Abdo. Pain 1/7 (14%) Nausea 4/7 (57%) Vomiting 3/7 (42%) Diarrhoea 1/7 (14%) **Constitutional** Fevers 4/7 (57%)	Pyrexia 42% Hypoxia (SpO_2_ < 95%) 85%	Bilateral GGO 71% Consolidation 42% Subpleural sparing 14%	-	-	Admitted 7/7 Intubated 1/7 Steroids 6/7
Maddock et al., 2019; USA [21]	6	**Respiratory** SOB 2/6 (33%) Cough 4/6 (66%) Wheeze 1/6 (16%) **GI** Abdo. Pain 3/6 (50%) Nausea 3/6 (50%) Vomiting 2/6 (33%) Weight loss 1/6 (16%) **Constitutional** Fevers 4/6 (66%) Sweats 3/6 (50%) Myalgia 4/6 (66%) Weakness 3/6 (50%)	Pyrexia (83%)	Bilateral infiltrates 100%	Eos 0.0–2.9 × 10^9^ CRP 20.4–30.7 ESR 60–128 mm/h	32–79% macrophages on BAL 25–75% LLMs	Admitted 6/6 Intubation 1/6 Antibiotics 5/6 Steroids 4/6
Schäfer et al., 2021; Germany [22]	1	SOB, dry cough, weight loss, fatigue	Hypoxia (SpO_2_ < 95%)	Bilateral GGO Interlobular septal thickening	Eos 0.1 × 10^9^ CRP 11.9 mg/dl	88% neutrophils on BAL	Admitted IV steroids
Adhikari et al., 2021; USA [23]	1	SOB, tachypnoea, nausea, diarrhoea, fever	Pyrexia Hypoxia (SpO_2_ < 95%)	Bilateral infiltrates	CRP 35 mg/dL ESR 97 mm/h	-	Admitted Antibiotics Steroids
Ganne et al., 2021; USA [24]	1	SOB, cough, fevers, myalgia, fatigue	Hypoxia (SpO_2_ < 95%)	Bilateral infiltrates	CRP > 400 mg/L	-	Admitted Antibiotics Steroids
Wekon-Kemeni et al., 2021; USA [25]	1	Nausea, vomiting, abdominal pain, fevers, headaches	Pyrexia Hypoxia (SpO_2_ < 95%)	Multifocal GGO, crazy paving	CRP 303 mg/L ESR 86 mm/h	-	Admitted Antibiotics Steroids
Guarino et al., 2021; Italy [26]	1	SOB, cough	-	Focal GGO, Consolidation, nodular change	CRP 0.4 mg/dLESR 17 mm/h	95% macrophages on BAL ORO stain positive	Admitted Antibiotics Steroids
Colesar et al., 2021; USA [27]	1	SOB, cough, chest pain, vomiting, fevers, headache	Pyrexia	Bilateral GGO	-	LLM identified on BAL cytology	Admitted Intubated Antibiotics IV steroids
O’Carroll et al., 2020; Ireland [28]	1	Cough, weight loss, sweats, fever	Pyrexia	Bilateral GGO Subpleural sparing	Eos 0.85 × 10^9^ CRP normal ESR 100 mm/h	66% macrophages on BAL 25% LLMs	Admitted
Wolf et al., 2020; USA [7]	1	SOB, sore throat, fevers	Hypoxia (SpO_2_ < 95%)	Bilateral nodular GGO Basal consolidation	Eos 5.8 × 10^9^	36% eosinophils on BAL	Admitted Intubated IV steroids
Bozkanat et al., 2020; USA [29]	1	SOB, cough, abdominal pain, diarrhoea, weight loss	Pyrexia Hypoxia (SpO_2_ < 95%)	Bilateral GGO and scattered opacities	CRP 33 mg/dL	-	Admitted Steroids
Jankharia et al., 2020; India [30]	1	Cough	-	Bilateral GGO and opacities	-	-	Antibiotics
Smith et al., 2020; USA [31]	1	SOB, chest pain, nausea, vomiting, diarrhoea, fevers, headache	Pyrexia	Bilateral GGO Subpleural sparing	CRP 30.56 mg/dL ESR 124 mm/h	-	Admitted Antibiotics
Matta et al., 2020; USA [32]	1	Nausea, vomiting, weight loss, fever, headache	Pyrexia	Diffuse patchy GGO Interlobular septal thickening Subpleural sparing	CRP 22.0 mg/dL ESR 46 mm/h	-	Admitted IV steroids
Thota et al., 2014; USA [8]	1	SOB, cough, facial flushing	-	Bilateral GGO Upper and middle lobe predominance	Eos 2%	74% eosinophils on BAL	Admitted Antibiotics Steroids

Abbreviations: # = number of patients; GI = gastrointestinal; GGO = ground-glass opacities; fibrotic features = reticulation, bronchiectasis, honeycombing; EO = eosinophil; LLM = lipid-laden macrophages.
